# The landscape and prognostic value of immune characteristics in uterine corpus endometrial cancer

**DOI:** 10.1042/BSR20202321

**Published:** 2021-04-22

**Authors:** Wenli Liu, Lisha Sun, Juan Zhang, Wengang Song, Mingcheng Li, Hong Wang

**Affiliations:** 1 Department of Gynecologic Oncology, The First Affiliated Hospital of Henan Polytechnic University, Jiaozuo 454000, China; 2 Clinical Laboratory Diagnostics, Medical Technology College, Beihua University, Jilin 132013, China

**Keywords:** checkpoint, endometrial cancer, ImmPort, immune cell, immune-related gene

## Abstract

In the present study, we explored the clinical and immunological characteristics of 575 uterine corpus endometrial carcinoma (UCEC) samples obtained from The Cancer Genome Atlas (TCGA) using the ESTIMATE and CIBERSORT algorithms. First, Kaplan–Meier and univariate Cox regression analyses indicated that the immune cell score was a prognostic factor for overall survival (OS) and recurrence-free survival (RFS). Multivariate Cox regression analysis further revealed that the immune cell score was an independent prognostic factor for UCEC patients. Second, we investigated the correlation between the infiltration levels of 22 types of immune cells and the immune score. Survival analysis based on the 22 immune cell types showed that higher levels of regulatory T cell, activated NK cell, and follicular helper T-cell infiltration were associated with longer OS, while higher levels of CD8+ T cell and naive B-cell infiltration were associated with longer RFS. Next, we performed differential expression and prognosis analyses on 1534 immune-related genes and selected five from 14 candidate genes to construct a prognostic prediction model. The area under the receiver-operating characteristic (ROC) curve (AUC) for 3- and 5-year survival were 0.711 and 0.728, respectively. Further validation using a stage I–II subgroup showed similar results, presenting AUC values for 3- and five-year survival of 0.677 and 0.692, respectively. Taken together, the present study provides not only a deeper understanding of the relationship between UCEC and the immune landscape but also guidance for the future development of UCEC immunotherapy.

## Introduction

Uterine corpus endometrial carcinoma (UCEC) is the seventh most commonly diagnosed cancer in women worldwide [1]. Approximately 75% of patients with endometrial cancer are diagnosed at an early stage, and local lesions can, to a large extent, be cured by surgery. Nevertheless, in some cases, radiation therapy is still needed after surgery. The 5-year overall survival (OS) rate in early-stage UCEC ranges from 74 to 91%. Chemotherapy and hormone therapy are viable treatment options for patients with metastasis or recurrence; however, not all patients benefit from these. For advanced stage III or IV disease, the 5-year OS rates are 57–66% and 20–26%, respectively [2]. Tumor cell growth requires the establishment of an immunosuppressive environment, such as the down-regulation of the expression of HLA-class I molecules and up-regulation of immunosuppressive factors, including PD1, PD-L1, TIM3, LAG3, and TIGIT, resulting in the absence of an effective immune system response [3–5]. In addition, several studies have shown that immune cell infiltration is significantly associated with prognosis in some tumors [6–9] and may be a promising source of new diagnostic and prognostic biomarkers.

In the present study, we systematically evaluated the prognostic value of immune cell infiltration and immune-related genes in UCEC. We first used the ESTIMATE algorithm to assess the impact of the immune score on clinical features and prognosis of UCEC. Subsequently, we utilized the CIBERSORT algorithm to evaluate 22 types of tumor-infiltrating immune cells, and further revealed a correlation between immune score and tumor-infiltrating immune cells. Following this, we explored the prognostic value of these cells. We also identified several immune-related genes involved in tumor development that could affect prognosis, and established a predictive model with five immune-related genes to assess disease prognosis and patient survival. This is the first and most comprehensive study concerning the clinical, molecular, and immunological characteristics of UCEC. We believe that this report will improve the understanding of the immune landscape in UCEC.

## Results

### The immune score was significantly associated with UCEC prognosis

**Figure 1:**
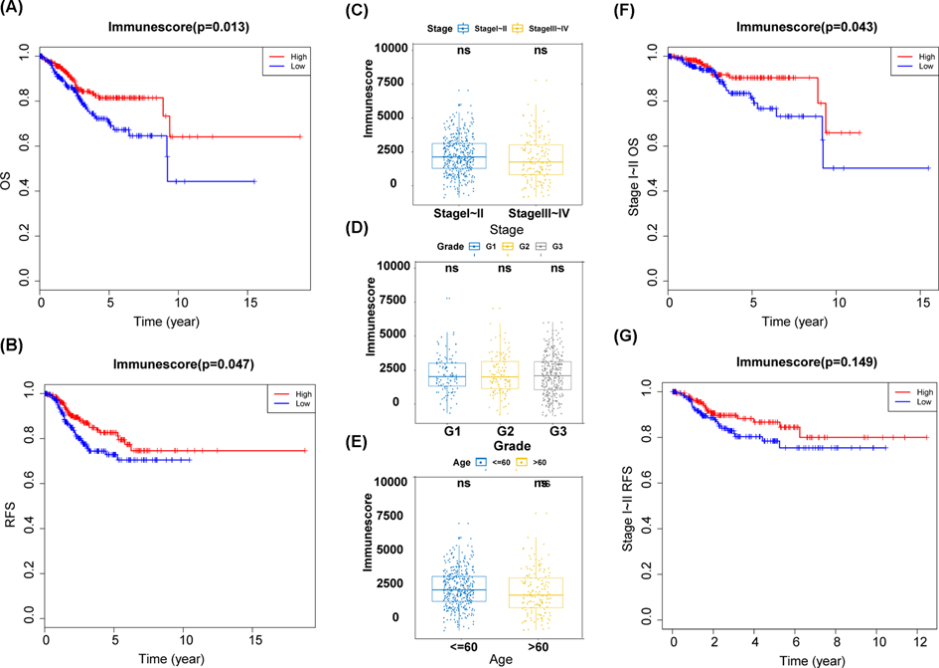
The immune score was significantly associated with UCEC prognosis (**A**) Kaplan–Meier curves for overall survival (OS) between the high and the low immune scores. (**B**) Kaplan–Meier curves for recurrence-free survival (RFS) between the high and the low immune scores. (**C**) The correlation between clinical stages and immune scores. (**D**) The correlation between clinical grades and immune scores. (**E**) The correlation between patients' age and immune scores. (**F**) Kaplan–Meier curves for OS between the high and the low immune scores at stages I and II. (**G**) Kaplan–Meier curves for RFS between the high and the low immune scores at stages I and II.

In the present study, we used the expression data and clinical information for 536 UCEC patients obtained from The Cancer Genome Atlas (TCGA) database. Based on the ESTIMATE algorithm, we calculated the immune score for each sample and used the median score as a cut-off to classify all patients into high-score and low-score groups. Kaplan–Meier survival curves indicated that patients with a high immune score had a better OS and recurrence-free survival (RFS) than those with a low immune score (Figure 1A,B). Importantly, univariate and multivariate Cox regression analysis based on TCGA dataset demonstrated that immune score was an independent prognostic factor in UCEC patients (Table 1). Our results further showed that there was no significant correlation between immune score and important clinical variables (Figure 1C–E), further supporting that the immune score was an independent prognostic factor.

**Table 1:** Univariate and multivariate regression analyses for predicting overall survival and recurrence-free survival of UCEC

	OS		RFS	
Characteristic	*P*	HR	*P*	HR
Univariate analysis				
Immunescore	0.0050	0.9998	0.0427	0.9998
Stage	<0.0001	1.8162	0.0036	1.9880
Grade	0.0493	3.9844	0.3162	1.1707
Age	0.0141	1.3621	0.2790	1.3040
Multivariate analysis				
Immunescore	0.0145	0.9998	0.0672	0.9998
Stage	<0.0001	4.7557	0.0133	2.0678
Grade	0.3962	1.1486	0.4838	1.1199
Age	0.2338	0.6645	0.5417	0.8301

Abbreviations: OS, overall survival; RFS, recurrence-free survival.

### The immune score was significantly associated with prognosis for patients with early-stage UCEC

Based on the International Federation of Gynecology and Obstetrics (FIGO) staging system, stage I and II UCECs are considered to be early-stage diseases. After surgery and adjuvant radiotherapy/chemotherapy, patients usually achieve relatively good outcomes [10–12]. In contrast, Stage III and IV UCECs are generally considered to be advanced diseases, and the OS is low even with multidimensional, high-intensity treatment. Considering the clinical importance of early UCEC diagnosis, we evaluated the prognostic value of the immune score for patients with stage I and II UCEC. The results showed that the immune score was also an important prognostic factor for OS (Figure 1F,G).

### The correlation between immune-infiltrating cells and the immune score in UCEC

**Figure 2:**
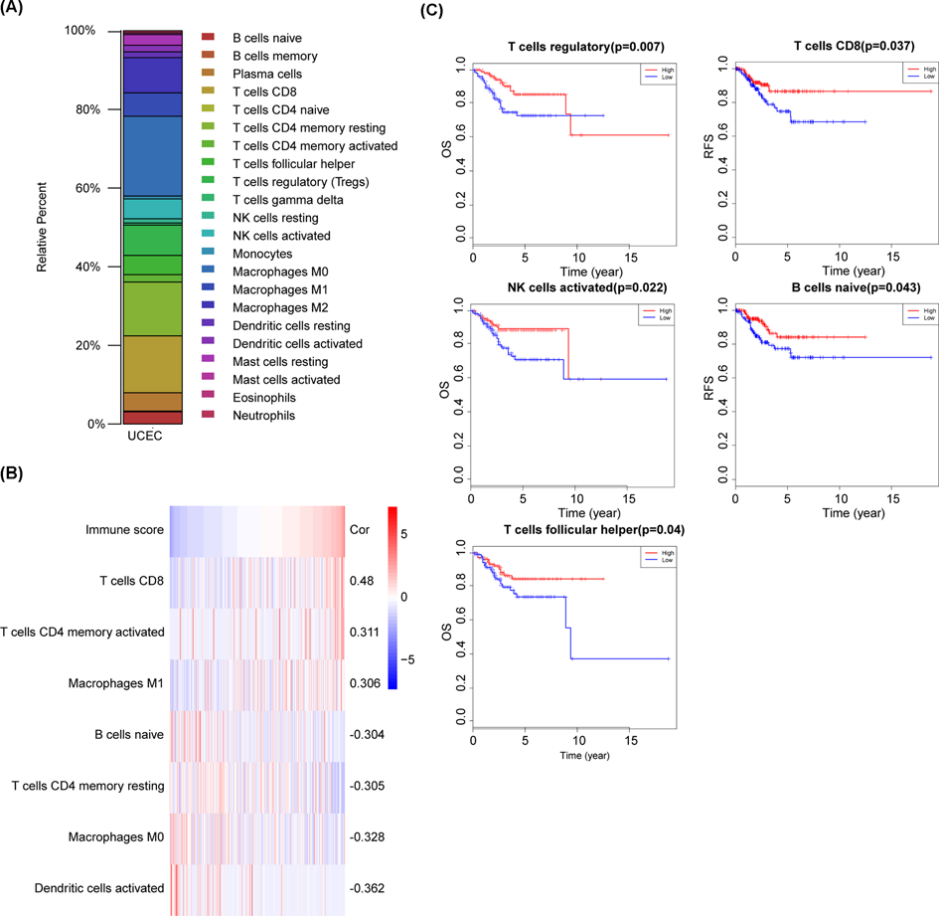
The correlation between immune-infiltrating cells and the immune score in UCEC and their prognostic value (**A**) The proportions of 22 types of tumor-infiltrating immune cells in uterine corpus endometrial carcinoma (UCEC). (**B**) Correlation between tumor-infiltrating immune cells and the immune score (|Cor >0.3|). (**C**) Kaplan–Meier curves for overall survival (OS) and recurrence-free survival (RFS) between high and low levels of immune cell infiltration.

Next, we used the CIBERSORT algorithm to estimate the proportions of 22 types of immune cells in UCEC (Figure 2A), and then evaluated the relationship between the immune score and the infiltration level of 22 immune cell types. The results showed that the immune score was positively correlated with CD8+ T cells, activated memory CD4+ T cells, and M1 macrophages, and negatively correlated with activated dendritic cells, M2 macrophages, resting memory CD4+ T cells, and naive B cells (Figure 2B).

### The prognostic value of tumor-infiltrating immune cells in UCEC

Given the importance of the immune score for OS and RFS, we evaluated the association between immune cell proportions determined with CIBERSORT and OS/RFS using Kaplan–Meier survival curves. We found that higher levels of regulatory T cells, activated NK cells, and follicular helper T cells were associated with better OS, and higher levels of CD8+ T cells and naive B cells were associated with better RFS (Figure 2C).

### The identification of differentially expressed immune- and prognosis-related genes

**Figure 3:**
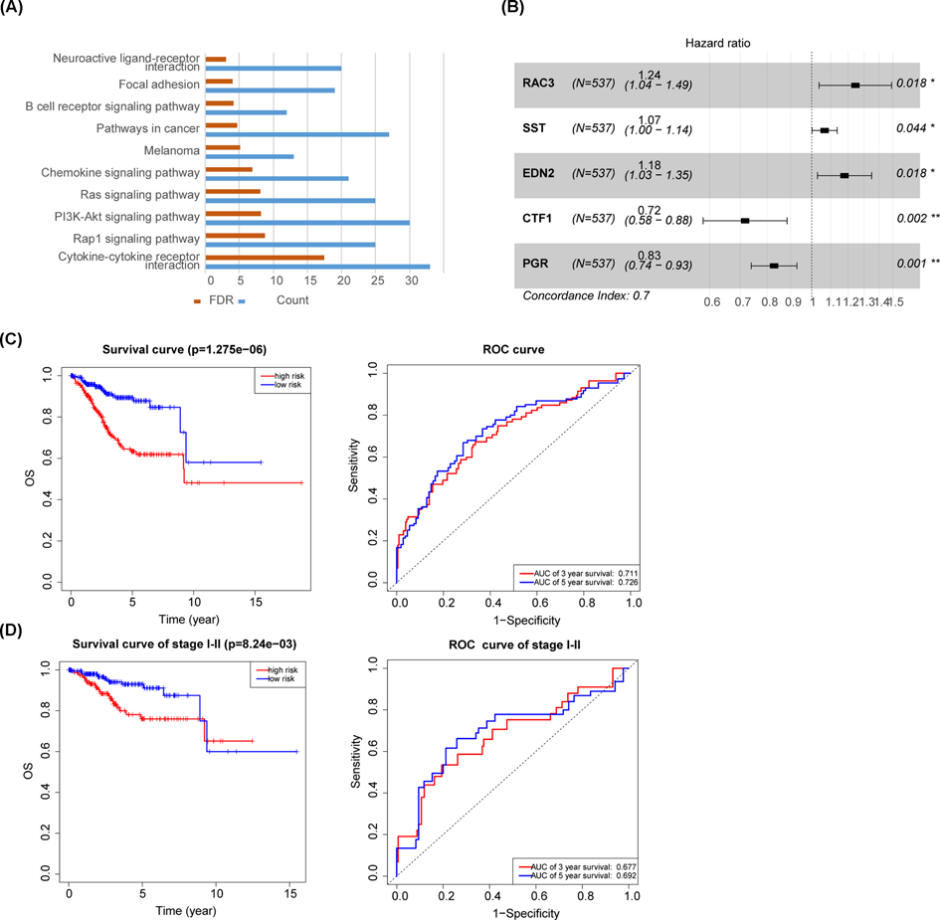
The construction of an immune-related five-gene signature (**A**) Kyoto Encyclopedia of Genes and Genomes (KEGG) annotation analysis of 186 differentially expressed immune-related genes. (**B**) Cox multivariate regression analysis of an immune-related five-gene signature for overall survival (OS). (**C**) Kaplan–Meier curves and Receiver–operating characteristic (ROC) curves for OS between high- and low-risk groups. (**D**) Kaplan–Meier curves and Receiver–operating characteristic (ROC) curves for OS between high- and low-risk groups for the stage I–II subgroups.

A total of 1534 genes downloaded from the ImmPort database were included in a differential gene expression analysis between 541 primary tumor samples and 35 normal samples (log FC >1 or log FC <−1). A total of 186 differentially expressed genes (DEGs) were identified, including 75 that were up-regulated and 111 that were down-regulated. Kyoto Encyclopedia of Genes and Genomes (KEGG) annotation analysis showed that the DEGs were mostly enriched in the cytokine–cytokine receptor interaction, Rap1, and PI3K/AKT signaling pathways (Figure 3A). These 186 DEGs were further included in a univariate Cox regression analysis. Combined with differential gene expression analysis, we identified five upregulated genes that were related to worse OS and nine downregulated genes that were related to better OS (Table 2).

**Table 2:** General characteristics of differentially expressed immune-related genes which were significantly correlated with prognosis

Gene	HR	*Z*-value	*P*-value	logFC	FDR
GDF7	0.651	-2.451	0.014	-1.506	<0.001
TNFSF12	0.712	-2.705	0.007	-1.674	<0.001
CTF1	0.756	-2.702	0.007	-1.333	<0.001
PGR	0.774	-4.669	0.000	-2.380	<0.001
ESR1	0.793	-4.424	0.000	-1.130	<0.001
ADCYAP1R1	0.795	-3.303	0.001	-1.240	<0.001
PTGER2	0.807	-1.999	0.046	-1.403	<0.001
NDP	0.813	-2.819	0.005	-2.115	<0.001
PTGDS	0.893	-2.074	0.038	-2.079	<0.001
SST	1.125	3.992	0.000	1.610	0.004
EDN2	1.160	2.226	0.026	1.519	<0.001
IL1RN	1.186	2.213	0.027	1.561	<0.001
RAC3	1.333	3.192	0.001	2.454	<0.001
GPI	1.424	2.453	0.014	1.253	<0.001

### The construction of an immune-related five-gene signature

Based on a multivariate Cox regression analysis of 14 important genes, we constructed a prognostic five-gene expression signature that included *RAC3, SST, EDN2, CTF1*, and *PGR* (Figure 3B). The risk score was calculated as follows: risk score = 0.2186 × RAC3 + 0.0647 × SST + 0.1640 × EDN2 – 0.3327 × CTF1 – 0.1880 × PGR. Patients were divided into high-risk and low-risk groups using the median risk score as a cut-off. Survival analysis based on the risk score could clearly discriminate the high-risk group from low-risk group (*P*<0.001). The AUC values for 3- and 5-year survival were 0.711 and 0.726, respectively (Figure 3C).

Given the importance of early treatment for a positive prognosis of UCEC, we selected the stage I–II subgroup to further validate the model and obtained similar results (*P*=0.008). The AUC values for 3- and 5-year survival in the model were 0.677 and 0.692, respectively (Figure 3D).

## Discussion

The past two decades have seen substantial progress in our understanding of human immune function. In particular, the identification of PD1 receptor inhibitors, followed by the identification of immune checkpoint blockers and immune checkpoint agonists, has led to a “golden age” of immunotherapy [13–15]. Furthermore, the importance of the function of immune cells in the tumor microenvironment (TME) has been increasingly recognized. It is now known that some TME-infiltrating immune cells play a role in tumor immune escape [16–18]. Therefore, in our study, we first used TCGA dataset to reveal the clinical features and immune landscape of UCEC.

We first assessed the prognostic value of the immune score and found that immune cell infiltrate levels in the TME were significantly associated with patient survival. We further analyzed the correlation between 22 immune cell types and the immune score and found that CD8+ T cells had the highest correlation with the immune score, indicating that they may play a key role in the UCEC microenvironment. Subsequently, we comprehensively analyzed the prognostic value of the 22 UCEC-infiltrating immune cell types and established that CD8+ T cell infiltration was closely associated with RFS, and that the high- and low-infiltration groups showed considerable stratification with regard to OS. This indicated that CD8+ T cells play a tumor-suppressive role, which is consistent with several previously reported findings [19–21]. NK cells are innate immune cells that not only have a strong lysis effect on abnormal or tumor cells but also act as regulators of the immune system. NK cells are associated with a good prognosis in several cancers, such as osteosarcoma [22] and gastric cancer [23]. Similarly, our results showed that NK cells are associated with longer OS in UCEC.

Regulatory T cells (Tregs) represent a class of lymphocytes functioning as host’s homeostasis by preventing the immune system activation toward self or harmless antigens [24]. The infiltration of Tregs in the tumor microenvironment suppresses the immune response against tumor-associated antigens, thus promoting tumor progression in non-small cell lung carcinoma (NSLC) [25], breast carcinoma [26] and melanoma, and are associated with a worse prognosis. In the tumor microenvironment, the release of cytokines and chemokines by dysplastic cells and tumor stroma infiltrating cells promote the accumulation of Tregs, thereby suppressing the anti-tumor activity mediated by NK cells and CD8+ T cells, finally accelerating tumor progression [27]. However, in sporadic colorectal cancer (CRC), Tregs infiltration plays dual roles on tumor progression and prognosis. The accumulation of Tregs among tumor infiltrating lymphocytes have been correlated with tumor stage and shorter disease-free survival, due to Tregs suppressive function against anti-tumor activity mediated by TAA-specific CD8+ cytotoxic cells [28]. In contrast, high number of Tregs in CRC stroma was associated with reduced tumor growth, indicating a better prognosis [29], because of Tregs suppressive function against pro-tumorigenic inflammation evoked by bacterial antigens influx in the tumor stroma. Interestingly, we found that regulatory T cells were beneficial for survival, which suggests that these cells may mediate different physiological functions in different tumor types. These discordant functions might stem from the heterogeneity of cells expressing FoxP3, their phenotypic plasticity and the role played by the unique bacteria-induced inflammatory microenvironment [28].

In addition, we also found that follicular helper T cells were associated with better outcomes. Follicular helper T cells promote the development of B-cell extracellular foci and germinal center antibody responses, which are essential for B-cell affinity maturation and the maintenance of humoral memory. Follicular helper T cells were also shown to prolong survival and reduce immunosuppression in squamous cell lung carcinoma [30]. Especially, according to previous studies, naive B cells are closely related to the severity of cancer, which fits the results of our study that the immune score was negatively correlated with naive B cells and naive B cells were associated with better RFS [31].

Next, we tested the expression and prognostic value of 1534 immune-related genes, and identified 14 DEGs that were closely associated with better OS. Based on Cox regression analysis, we selected five key genes—*RAC3, SST, EDN2, CTF1*, and *PGR*—to construct a prognostic prediction model. *RAC3* is considered to function as an oncogene and was originally found to be highly expressed in breast cancer [32]. Subsequent studies showed that *RAC3* was involved in the regulation of the cell cycle, cell proliferation [33,34], apoptosis, the inhibition of autophagy [35,36], cell migration, and epithelial–mesenchymal transition (EMT) [37,38], all of which are closely related to tumor development. The SST (somatostatin) protein functions as a tumor suppressor in various tumor types through binding to somatostatin receptors [39,40]. Studies have shown that *EDN2* is highly expressed in clear cell renal cell carcinoma, cervical cancer, and breast cancer [41–43]. Contradictory results have been reported for the role of *EDN2* in cancer. Interestingly, high *EDN2* expression appears to be associated with both better survival in renal cell carcinoma patients and the promotion of the invasive abilities of breast cancer cells. Our results support its status as an oncogene. CTF1 induces the expression of IL-6 in epithelial cells, endothelial cells, and monocytes, thereby exerting anti-inflammatory, proinflammatory, and cytoprotective effects [44]. Currently, research into this molecule is less involved in the field of tumors. Therefore, the anticancer effect of CTF1 identified in our study suggests that this molecule may have a role in the field of cancer treatment. The progesterone receptor (PGR) is widely documented as playing a role in various gynecological tumors. Single-nucleotide polymorphisms (SNPs) in the *PGR* gene have been associated with a risk of UCEC [45]. Here, we explored the relationship between PGR transcript levels and endometrial cancer, and found that elevated *PGR* expression was associated with better prognosis in UCEC patients. Further studies on PGR protein levels and the mechanisms underlying PGR effects in UCEC are required in the future.

Our data were obtained from TCGA database and comprised a large patient sample and diverse data types. Nevertheless, this was still a retrospective study, and prospective studies are needed to validate these results.

Taken together, our analysis of immune infiltration and immune-related genes provides not only a deeper understanding of the relationship between UCEC and the immune landscape but also guidance for the future development of UCEC immunotherapy.

## Materials and methods

### Data mining from TCGA dataset

Expression and clinical data for 575 UCEC samples, including age, grade, stage, and prognostic information, were extracted from TCGA Genome Data Analysis Center (https://cancergenome.nih.gov). The samples comprised 541 primary tumor samples and 35 normal samples. Of the 575 samples, 536 contained complete survival data. A set of 1534 immune-related genes were downloaded from the ImmPort database (https://immport.niaid.nih.gov).

### Evaluation of tumor-infiltrating immune cells using ESTIMATE and CIBERSORT algorithms

To estimate the immune cell landscape in each tumor, endometrial cancer data from TCGA database were analyzed using the ESTIMATE and CIBERSORT algorithms. ESTIMATE analyzes tumor expression data to predict the tumor stromal score [46]. The stromal score measures interstitial cell infiltration. CIBERSORT was used in combination with the LM22 signature matrix to quantify the relative levels of 22 immune cell types in complex gene expression mixtures, including resting memory CD4+ T cells, naive CD4+ T cells, CD8+ T cells, follicular helper T cells, gamma delta T cells, activated memory CD4+ T cells, regulatory T cells, memory B cells, naive B cells, activated dendritic cells, resting dendritic cells, eosinophils, macrophages (M0–M2), activated mast cells, resting mast cells, monocytes, activated NK cells, resting NK cells, neutrophils, and plasma cells. Only patients with CIBERSORT-derived *P*-values <0.05 were included in this analysis [47].

### Statistical analysis

Survival curves were estimated using the Kaplan–Meier method and compared using the log-rank test. Cox regression analysis was used to identify independent prognostic factors and establish predictive models. The ROC curve was used to further assess the accuracy of the model predictions. Statistical analyses were performed using SPSS version 21.0 (SPSS, Chicago, IL, U.S.A.) and R software version 3.5.2. For all statistical analyses, *P*-values <0.05 were considered significant.

## Data Availability

All supporting data are included within the main article and its supplementary files.

## Abbreviations

AUC: area under the ROC curve
DEG: differentially expressed gene
FC: fold change
FIGO: Federation of Gynecology and Obstetrics
KEGG: Kyoto Encyclopedia of Genes and Genomes
OS: overall survival
RFS: recurrence-free survival
ROC: receiver-operating characteristic
SNP: single-nucleotide polymorphism
TCGA: The Cancer Genome Atlas
TME: tumor microenvironment
UCEC: Endometrial cancer of the uterine corpus
